# The complete mitochondrial genome of the nematode-trapping fungus *Dactylellina haptotyla*

**DOI:** 10.1080/23802359.2018.1507650

**Published:** 2018-10-30

**Authors:** Duanyong Zhou, Yunrun Zhang, Jianping Xu, Lili Jiang, Ke-Qin Zhang, Ying Zhang

**Affiliations:** a State Key Laboratory for Conservation and Utilization of Bio-Resources in Yunnan, and Key Laboratory for Southwest Microbial Diversity of the Ministry of Education, Yunnan University, Kunming, Yunnan, P. R. China;; b School of Life Science, Yunnan University, Kunming, Yunnan, P. R. China;; c Department of Biology, Mc Master University, Hamilton, Canada

**Keywords:** *Dactylellina haptotyla;* mitogenome, phylogenetic analyses

## Abstract

The complete mitochondrial genome of *Dactylellina haptotyla* was studied in this study. This mitogenome is a closed circular molecule of 146,101 bp in length with a GC content of 22.92%, including 14 protein-coding genes, 26 transfer RNA genes, 2 ribosomal RNA genes. Phylogenetic analyses based on sequences at the 14 concatenated mitochondrial protein-coding genes showed that *D. haptotyla* was closely related to *Pyronema omphalodes.*

*Dactylellina haptotyla* (Ascomycota, Orbiliomycetes, Orbiliales, Orbiliaceae) is a nematode-trapping fungus within the group of soil-living fungi found worldwide, which has the infection structures called knobs to capture and kill nematodes (Drechsler 1950). Recently, a large number of genes were found highly expressed and differentially regulated during nematode infection in this fungus (Meerupati et al. 2013). However, little is known about its mitogenome. Here, we report the complete mitogenome of *D. haptotyla* and investigate the phylogenetic relationships with other related species based on mitochondrial proteins.

The mitogenome was extracted from the whole genome sequence of a pure culture of strain CBS 200.50 (collected from decaying leaf in pond, at Chelsea Physic Garden, London, by M.P. Peach, 1948. N 51°29′ E 0°9′.). We downloaded the genome sequence of *D. haptotyla* strain CBS 200.50 (GenBank ID: AQGS00000000.1) and annotated the complete mitochondrial genome with *Pyronema omphalodes* (GenBank ID: KU707476) as a reference. The mitogenome was identified by BLAST as scaffold00324 and scaffold00325 (GenBank: KE525747.1, KE525748.1, respectively) in whole genome and annotated using MFannot (http://megasun.bch.umontreal.ca/cgi-bin/mfannot/mfannotInterface.pl) and GLIMMER (https://www.ncbi.nlm.nih.gov/genomes/MICROBES/glimmer_3.cgi). tRNAs were annotated using tRNAscan-SE (Schattner et al. 2005). All ORFs were searched and identified by ORF Finder. The complete mitogenome of *D. haptotyla* is a closed circular molecule of 146,101** **bp in length with a GC content of 22.92%, it consists of 14 core mitochondrial protein-coding genes, 26 tRNA genes, 2 rRNA genes (small and large subunit rRNA). Protein-encoding genes include three ATP synthase subunits (atp6, atp8 and atp9), three cytochrome oxidase subunits (cox1, cox2 and cox3), one apocytochrome b (cob), and seven NADH dehydrogenase subunits (nad1, nad2, nad3, nad4, nad4L, nad5 and nad6). All the predicted genes totally possessed 27 introns, but none of the tRNAs possessed introns. All tRNA genes are encoded on the sense strand and covered 20 kinds of standard amino acids.

Phylogenetic analysis of *D. haptotyla* was performed by comparison with 14 mitochondrial proteins of other 20 species in Ascomycota was performed by Bayesian inference (BI). As shown in Figure 1. *Dactylellina haptotyla* was a member of Ascomycota and closely related to *P. omphalodes.* The phylogenetic relationship using mitochondrial proteins were in accordance with those constructed based on sequences of nuclear genes (Sugiyama et al. 2006). The Genbank Accession number for Dactylellina haptotyla is MK554671.

**Figure 1.**
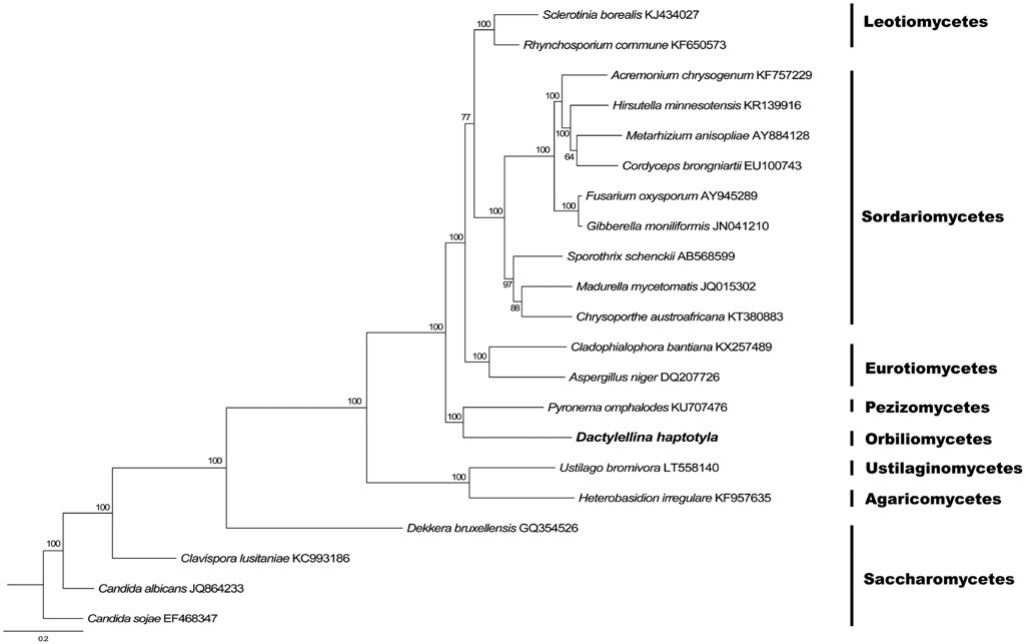
Phylogenetic relationships among 20 Ascomycota fungi inferred based on the concatenated amino acid sequences of 14 mitochondrial protein-coding genes. The 14 mitochondrial protein-coding genes were: nad1, nad2, nad3, nad4, nad4L, nad5, nad6, cox1, cox2, cox3, cob, atp6, atp8, atp9. The tree was generated using Bayesian inference (BI). Numerical values along branches represent statistical support based on 1000 randomizations.
